# Leveraging Deep Learning Decision-Support System in Specialized Oncology Center: A Multi-Reader Retrospective Study on Detection of Pulmonary Lesions in Chest X-ray Images

**DOI:** 10.3390/diagnostics13061043

**Published:** 2023-03-09

**Authors:** Daniel Kvak, Anna Chromcová, Robert Hrubý, Eva Janů, Marek Biroš, Marija Pajdaković, Karolína Kvaková, Mugahed A. Al-antari, Pavlína Polášková, Sergei Strukov

**Affiliations:** 1 Carebot, Ltd., 128 00 Prague, Czech Republic; 2 Faculty of Nuclear Sciences and Physical Engineering, Czech Technical University, 115 19 Prague, Czech Republic; 3 Department of Radiology, Masaryk Memorial Cancer Institute, 602 00 Brno, Czech Republic; 4 Faculty of Mathematics and Physics, Charles University, 121 16 Prague, Czech Republic; 5 Faculty of Electrical Engineering, Czech Technical University, 166 36 Prague, Czech Republic; 6 Department of Artificial Intelligence, Daeyang AI Center, Sejong University, Seoul 050 06, Republic of Korea; 7 Department of Imaging Methods, Motol University Hospital, 150 06 Prague, Czech Republic; 8 Department of Radiodiagnosis, Podripska City Hospital, 413 01 Roudnice nad Labem, Czech Republic

**Keywords:** convolutional neural network, computer-aided diagnosis, deep learning, object detection, lung cancer, pulmonary lesion, YOLO

## Abstract

Chest X-ray (CXR) is considered to be the most widely used modality for detecting and monitoring various thoracic findings, including lung carcinoma and other pulmonary lesions. However, X-ray imaging shows particular limitations when detecting primary and secondary tumors and is prone to reading errors due to limited resolution and disagreement between radiologists. To address these issues, we developed a deep-learning-based automatic detection algorithm (DLAD) to automatically detect and localize suspicious lesions on CXRs. Five radiologists were invited to retrospectively evaluate 300 CXR images from a specialized oncology center, and the performance of individual radiologists was subsequently compared with that of DLAD. The proposed DLAD achieved significantly higher sensitivity (0.910 (0.854–0.966)) than that of all assessed radiologists (RAD 10.290 (0.201–0.379), *p* < 0.001, RAD 20.450 (0.352–0.548), *p* < 0.001, RAD 30.670 (0.578–0.762), *p* < 0.001, RAD 40.810 (0.733–0.887), *p* = 0.025, RAD 50.700 (0.610–0.790), *p* < 0.001). The DLAD specificity (0.775 (0.717–0.833)) was significantly lower than for all assessed radiologists (RAD 11.000 (0.984–1.000), *p* < 0.001, RAD 20.970 (0.946–1.000), *p* < 0.001, RAD 30.980 (0.961–1.000), *p* < 0.001, RAD 40.975 (0.953–0.997), *p* < 0.001, RAD 50.995 (0.985–1.000), *p* < 0.001). The study results demonstrate that the proposed DLAD could be utilized as a decision-support system to reduce radiologists’ false negative rate.

## 1. Introduction

Chest X-ray (CXR) is one of the most common diagnostic imaging tests. It is often used to identify and monitor various chest findings, including pulmonary lesions that may be indicative of, among other pathologies, lung cancer. However, the effectiveness of X-ray imaging in detecting both primary and secondary tumors is not always reliable [1]. In addition, CXR is prone to reading errors and low interobserver and intraobserver agreements due to its limited spatial resolution and variability in human anatomic structures.

A pulmonary lesion can usually be observed as a rounded, well-defined opacity on CXR. The margins may be smooth or convoluted, and it is surrounded by normally aerated lung tissue. A lesion is defined by its size, location, margins, and density. This description plays an important role in the differential diagnosis of the particular patient, as well as the information about the number of lesions present since the background of a solitary pulmonary nodule, pulmonary mass, and multiple lesions may vary. Generally, if the lesion is less than 30 mm in diameter, it is considered a nodule; if more than 30 mm, it is considered a mass [2,3]. Occasionally, nodules as small as 5-6 mm in size can be visible on CXR [4]. Despite this, even larger lesions can remain undetected by a well-experienced radiologist; e.g., in [5], repeated CXR was used as a screening method for the detection of lung cancer. It ws found that 45 out of 50 carcinomas detected by the screening, all of which were more than 10 mm in diameter, could also be seen on the previous CXR images, which were originally misdiagnosed as normal CXRs.

Although most solitary pulmonary nodules are benign findings, when a lesion is discovered, the probability of its malignancy must be assessed using clinical and radiological features [6]. Clinical factors, which may increase the probability of malignity, include the size of the pulmonary lesion, age of the patient, history of the oncological disease, chronic tobacco smoking, asbestos exposure, or presence of other diseases such as chronic obstructive pulmonary disease [7]. A solitary pulmonary nodule less than 7 mm in diameter has a risk of malignant potential less than 1%, an 8–20 mm nodule’s risk is 18%, and nodules of 20–30 mm have a malignancy risk of 50% [8]. Moreover, a tumor of the lung does not always appear as a nodule or mass on a CXR. Tumors causing bronchial obstruction with atelectasis are difficult to distinguish from obstructive atelectasis due to other causes [9,10]. Centrally located tumors often cause mediastinal lymphadenopathy, and bronchial obstruction with atelecatsis, may spread directly into the mediastinum, and often have seeding infection that may mask the lesion [11]. In such cases, a tumor is suspected only on repeat imaging after pneumonia has healed [12].

A solitary pulmonary nodule has a wide differential diagnosis. Causes other than malignant tumors include benign tumors, inflammatory lesions (granuloma, abscess, rheumatoid nodule, inflammatory pseudotumor, round pneumonia), congenital processes (arteriovenous malformation, pulmonary cyst, bronchial atresia with mucous plug), and others (pulmonary infarction, intrapulmonary lymphatic nodule, mucous plug, pulmonary hematoma, pulmonary amyloidosis, confluention of the vessels). The diagnostic process is focused primarily on the differentiation between benign and malignant causes [13]. If the lesion is assessed as benign, the approach depends mainly on the patient’s clinical symptoms. Some pathologies only require to be observed by follow-up CXRs or CTs, and the pathology origin needs to be clarified in detail to correctly assess the potential risk to the patient and to set up the most beneficial treatment plan [14]. Pulmonary masses differ in size from pulmonary nodules, the arbitrary threshold being 30 mm. Primary lung tumors are the most common cause; a solitary lesion larger than 30 mm should be considered malignant unless proven otherwise [15]. However, as with other lesions, the differential diagnosis is very extensive, and the most common benign cause is granuloma (caused by sarcoidosis, infection, vasculitis, or rheumatoid disease).

**Figure 1:**
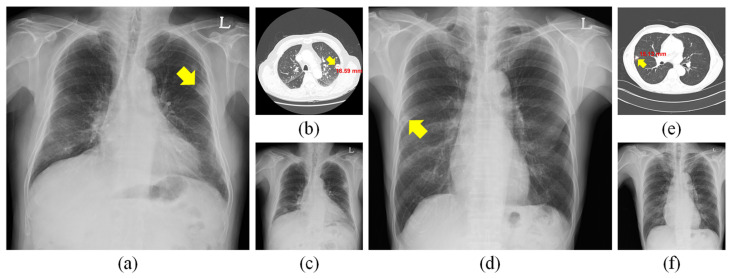
Initial and follow-up CXRs and CT images with a pulmonary lesion. (**a**) Initial CXR of a 65-year-old male patient with metastatic renal cell carcinoma in the left upper lobe and (**b**) CT examination of the patient (**a**). (**c**) Follow-up CXR of (**a**). (**d**) Initial CXR of an 81-year-old male patient with metastatic adenocarcinoma in the right middle lobe and (**e**) CT examination corresponding to (**d**). (**f**) Follow-up CXR of (**d**). The yellow arrows indicate the localization of suspected pulmonary lesions [16].

If a suspicion of lung cancer arises from the CXR, a contrast-enhanced chest CT is used for further assessment of the pathology’s nature and origin, and, in the case of a malignant tumor, for staging (Figure 1). CT shows the detailed location of the tumor, the pattern of its spread, and eventually, satellite foci, mediastinal and hilar lymphadenopathy, and contralateral lung metastases; if the epigastrium is also imaged, metastases to the liver or adrenal glands can be assessed. Recently, the role of CT has also been applied in the screening of selected patient groups: a lung cancer screening program has been running in the Czech Republic since January 2022.

The aim of this study is to demonstrate the effectiveness of the proposed DLAD for detecting pulmonary lesions on CXR images and to compare its performance with that of radiologists with different levels of experience in a simulated clinical setting. For this purpose, we performed a multi-reader, single-site, retrospective study.

## 2. Related Works

**Figure 2:**
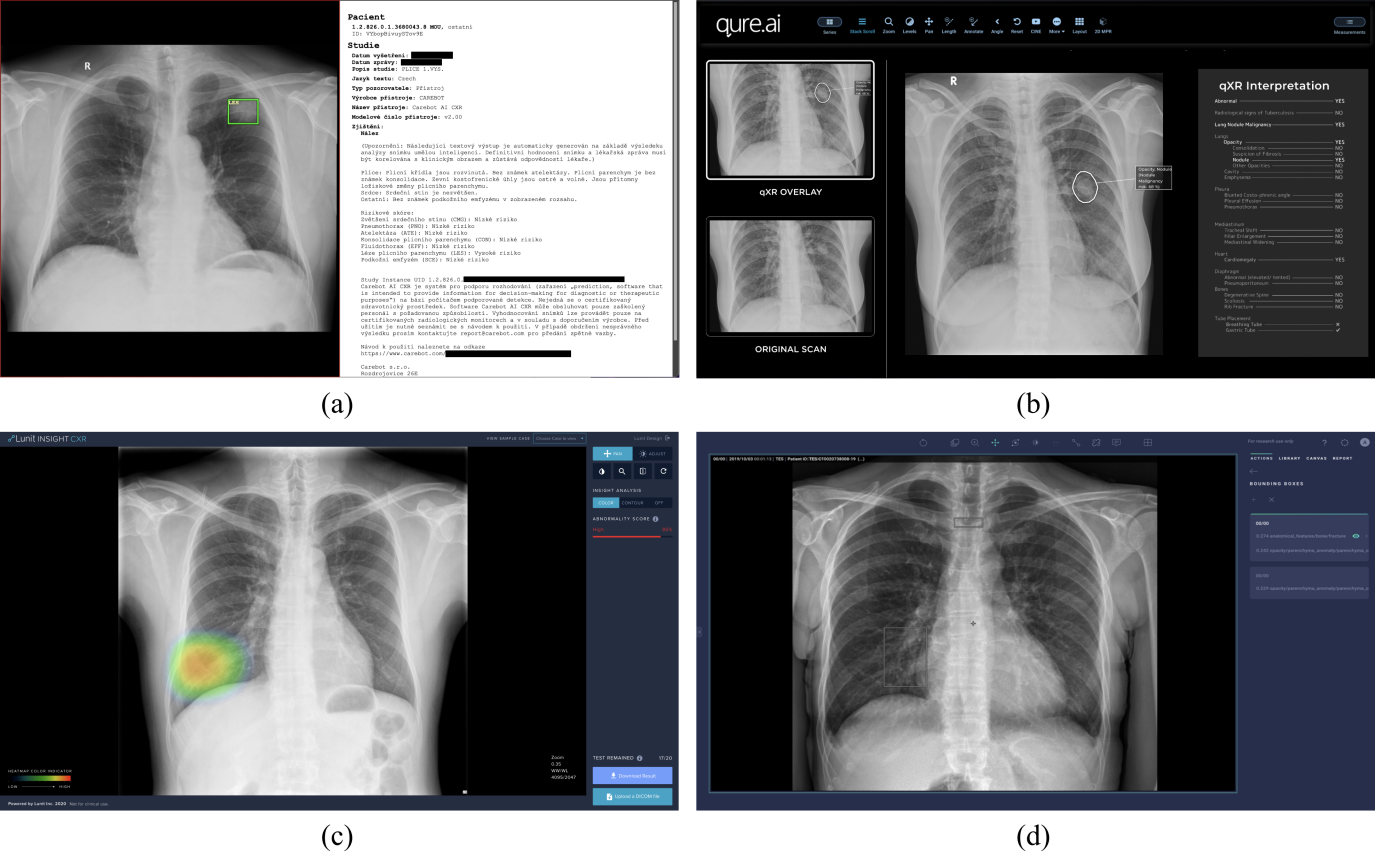
(**a**) The proposed DLAD (Carebot AI CXR v2.00, implemented in CloudPACS by OR-CZ) and other commercial solutions: (**b**) Qure AI qXR, (**c**) Lunit INSIGHT CXR, and (**d**) Arterys Chest AI.

Detection of pulmonary lesions on CXRs using DLAD is an active area of research in both commercial applications (Figure 2) and academic projects [17,18,19]. Due to the known limitations in transparency [20] and the lack of clinical relevance of publicly available datasets [21], we decided to exclude studies that leveraged these data to train or evaluate their DLAD in our analysis.

In [22], a commercial DLAD for detecting pulmonary lesions (*Lunit INSIGHT CXR*) was developed and tested on a dataset of CXRs. The DLAD performance was compared to that of 18 doctors. The investigated DLAD demonstrated a specificity (*Sp*) of 0.952 while preserving a high sensitivity (*Se*) of 0.807, whereas the average *Se* of the radiologists was 0.704. Ref. [23] evaluated the effectiveness of a commercial DLAD (*Riverain ClearRead Xray*) in improving the detection of pulmonary nodules on CXRs by radiologists. The DLAD was used to process 300 CXRs and generated bone-suppressed images (BSIs). Five radiologists and three residents evaluated the CXRs with and without the algorithm. The results showed that the DLAD achieved a *Se* of 0.74 and significantly improved the average performance of the radiologists, as measured by the area under the curve (*AUC*), from 0.812 to 0.841. The DLAD also detected 127 of the 239 nodules that were missed by the radiologists. In [24], the standalone performance of a commercial DLAD (*Arterys Chest AI*) was evaluated. The DLAD was tested on CXR data from a single hospital, and its performance in detecting opacities, pleural effusions, pneumothorax, nodules, and fractures was assessed. The study found that the algorithm achieved good *Se* and *Sp* in detecting opacities, pleural effusions, and pneumothorax but showed limited performance in detecting nodules and fractures due to the small number of these abnormalities in the dataset (24 nodules and 4 fractures). The *AUC* for the different pathologies ranged from 0.773 for pulmonary nodules to 0.991 for pneumothorax. Ref. [25] evaluated the effectiveness of commercial DLAD (*Samsung Electronics Auto Lung Nodule Detection*) in improving the detection of pulmonary lesions on CXRs by radiologists. The DLAD was tested on a dataset of 600 CXRs containing lung cancer and 200 normal CXRs from four medical centers. The performance of the DLAD was compared to that of 12 radiologists. The results showed that the DLAD had a significantly higher *Se* in detecting pulmonary lesions than the radiologists alone (0.86 versus 0.79) and also had a significantly higher *AUC* (0.88 versus 0.82). Ref. [26] evaluated the effectiveness of commercial DLAD (*Siemens Healthineers AI-Rad Companion*) in detecting pulmonary nodules on CXRs. The study included 100 CXR images from two centers in Germany and USA. The images were selected to represent nodules with different levels of detection difficulty. Nine radiologists from Germany and the US reviewed all of the images in two sessions, one with the DLAD-aided interpretation and one without, with a washout period in between. The study found that the mean detection accuracy for the radiologists improved by 6.4% with DLAD-aided interpretation compared to unaided interpretation. Junior radiologists saw a greater improvement in *Se* for nodule detection with AI-aided interpretation compared to senior radiologists, while senior radiologists experienced a similar improvement in *Sp* to junior radiologists. Ref. [27] leveraged a commercial DLAD (*Qure AI qXR*) in detecting malignant nodules on CXR images. The researchers compared the accuracy of DLAD to that of two radiologists in detecting nodules on 894 preselected CXRs. The results showed that DLAD had a high *AUC* of 0.99 and a *Se* of 1 at the operating threshold, indicating a high level of accuracy.

## 3. Materials and Methods

### 3.1. Software

Deep learning (DL) algorithms are a type of artificial intelligence (AI) that uses multi-layered artificial neural networks (ANN) to analyze and recognize patterns in data. Computer vision algorithms involve training robust convolutional neural networks (CNN) on large numbers of images. The CNNs are able to learn and make predictions based on the visual data and ground truth labels provided and have been shown to be particularly effective in the field of medical imaging [28,29].

The proposed DLAD (Carebot AI CXR v2.00) utilizes various deep learning techniques to identify and localize suspicious lesions in CXR images. It is designed to assist radiologists in interpreting CXRs in posterior–anterior (PA) or anterior–posterior (AP) projections. To maintain the existing user workflow, the DLAD was implemented into the picture-archiving and communication system (PACS) for potentially more efficient clinical deployment. The DLAD leverages DL algorithms to automatically detect abnormalities based on visual patterns for the following abnormalities: atelectasis (ATE), consolidation (CON), cardiomegaly (CMG), pneumothorax (PNO), pulmonary lesion (LES), pleural effusion (EFF), and subcutaneous emphysema (SCE). This study investigates solely the software module for identifying suspicious pulmonary lesions. Standardized descriptions of individual findings were determined through discussion with the radiologists involved in the development of the proposed DLAD. Detailed information can be found in the user manual provided to collaborating hospitals.

#### 3.1.1. Training Data

The development of the proposed DLAD involved 25,374 anonymized X-ray images with established ground truth from sites across Europe, Asia, and North America. The inclusion of CXRs from multiple hospitals was intended to account for the variation in screening quality [30] and local population characteristics [31]. The dataset excluded images of poor quality or incorrect projection, as well as those from patients under 18 years of age. A team of 27 radiologists, with varying levels of experience, annotated the collected CXR images. The final training dataset contained 12,149 images of patients with visually confirmed pathological findings and 13,225 images with either no or insignificant findings.

#### 3.1.2. Model Architecture

**Figure 3:**
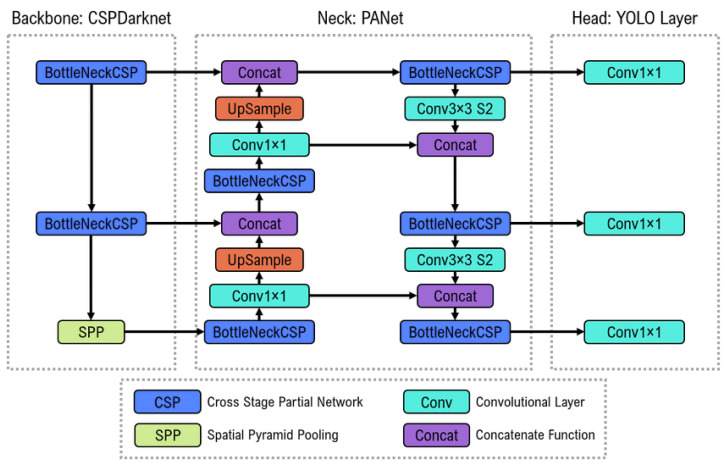
Overview of the YOLOv5 model architecture [34].

To develop the LES model for detecting suspicious lesions on CXR, the YOLOv5 architecture (Figure 3) was used. The main advantage of using an object-detection approach is the ability to accurately detect and localize even smaller pulmonary nodules that conventional convolutional neural networks may struggle with. Ref. [32] introduced YOLO, a novel approach to object detection, in 2016. The basic idea is dividing the input image into a grid of cells and predicting bounding boxes and class probabilities for each cell [33]. The bounding box coordinates are predicted as offsets relative to a set of anchor boxes, which are pre-defined default boxes with different aspect ratios. The YOLO model consists of three main components: a feature extractor, a predictor, and a postprocessor. The feature extractor is responsible for extracting relevant features from the input image, which are then passed through the predictor to produce the bounding box predictions. The postprocessor filters the predicted bounding boxes using non-max suppression to remove overlapping boxes and low-confidence detections.

#### 3.1.3. Communication Protocol

The proposed DLAD takes the form of a web-based application entity (AE) for the analysis of image documentation (DICOM CR, DX). It uses the DICOMweb™ standard to communicate with the connected PACS. The software takes the form of a prediction algorithm with application peripherals (web-based communication tools, DICOM file conversion, storage, and reporting libraries in proprietary DICOM Basic Text Structured Report and DICOM Presentation State formats).

### 3.2. Data Collection

To collect the data for this retrospective study, we addressed a specialized oncology center. Masaryk Memorial Cancer Institute is a leading cancer research and treatment hospital in the Czech Republic. A total of 9276 CXR images were retrieved from the period between June 2020 and July 2021. Data excluded images of children under 18 years of age, scans with technical issues, and images taken in lateral projection.

### 3.3. Ground Truth

To determine the ground truth for the presence or absence of a pulmonary lesion on CXR, we proposed that three radiologists with different levels of experience would be assessed for the initial reading. The evaluation included (A) a radiologist with less than 5 years of experience, (B) a board-certified radiologist with more than 5 years of experience, and (C) a head of the radiology department with more than 10 years of experience. At least 2 out of 3 radiologists needed to agree on the presence of a pulmonary lesion in order to establish the ground truth (Table 1). If there was a further disagreement between the assessed radiologists, the CXR was not included in the assessment. Subsequent pixel-level annotation was carried out by a board-certified head of the radiology department of a municipal hospital.

### 3.4. Assessment

A total of 300 CXR images were randomly selected for the experiment using the *random.choice()* function, of which 100 (55 ♀, 45 ♂) were from patients with a total of 385 confirmed pulmonary lesions (LES+ Abnormal), 100 (68 ♀, 32 ♂) were from patients with various confirmed findings other than pulmonary lesions (LES− Abnormal), and 100 (79 ♀, 21 ♂) were without any pathological findings (Normal). The prevalence of individual findings can be found in Table 2. Five independent radiologists were invited to evaluate the presence or absence of pulmonary lesions in the selected CXR images without being given any information about the patient’s history or previous or follow-up examinations. This was in order to objectively compare the results with those produced by the DLAD software. The experience levels of the radiologists were as follows: RAD 1 and RAD 2 were junior radiologists with less than 2 years of experience, RAD 3 was a radiologist with more than 2 years but less than 5 years of experience, and RAD 4 and RAD 5 were board-certified radiologists with more than 5 years of experience. Evaluation of CXR was conducted in Carebot Label App software, with each radiologist identifying if the CXR showed patterns for any of the 13 preselected abnormalities (atelectasis, consolidation, cardiomegaly, mediastinal widening, pneumoperitoneum, pneumothorax, pulmonary edema, pulmonary lesion, bone fracture, hilar enlargement, subcutaneous emphysema, and pleural effusion). The study was designed as blinded since the assessed radiologists were not aware of the ratio of lesion-positive or negative CXRs.

### 3.5. Statistical Analysis

The standard approach to measuring the performance in the detection of pulmonary lesions on CXR images is to use the measures of balanced accuracy (*BA*), sensitivity (*Se*), specificity (*Sp*), and positive *(PLR)* and negative likelihood ratio *(NLR)*. *Se* is the proportion of actual positive cases that are correctly identified as positive by the diagnostic test; e.g., it measures the proportion of true positive (TP) and false negative (FN) results. On the other hand, *Sp* is the proportion of actual negative cases that are correctly identified as negative by the diagnostic test; e.g., it measures the proportion of true negative (TN) and false positive (FP) results. The mutual relations are expressed by *PLR = Se/(1−Sp)* and *NLR = (1−Se)/Sp*. The likelihood ratios *(LRs)* depend only on *Se* and *Sp* and are equivalent to the relative risk. Higher *PLR* and lower *NLR* are desirable.

A paired design was applied to the data; i.e., each CXR was evaluated by DLAD and all assessed radiologists, and their evaluations were compared to the ground truth. To compare the *Se* and *Sp* of the DLAD with that of individual radiologists, we calculated and compared statistical parameters using confidence intervals (*CI*) and *p*-values. The null hypothesis test ($H_{0}$) was conducted to determine if there were significant differences between the DLAD and the radiologists. The procedure to compare the statistics consisted of (i) solving $H_{0}$ to an α error, calculating the Wald $\chi^{2}$ test (e.g., $H_{0}:(Se_{1}=Se_{2}$ *and* $Sp_{1}=Sp_{2})$ *vs.* $H_{1}:(Se_{1}\neq Se_{2}$ *and/or* $Sp_{1}\neq Sp_{2})$), and (ii) if $H_{0}$ was rejected, conducting alternative hypothesis test ($H_{1}$) with a multiple comparison method (e.g., McNemar with continuity correction for *Se* and *Sp* and the Holm method for *LRs*) to an α error. Forest plots were used to visualize the differences in *Se* and *Sp* between individual radiologists and DLAD. *CIs* were constructed at a two-tailed 95% confidence level.

## 4. Results

**Figure 4:**
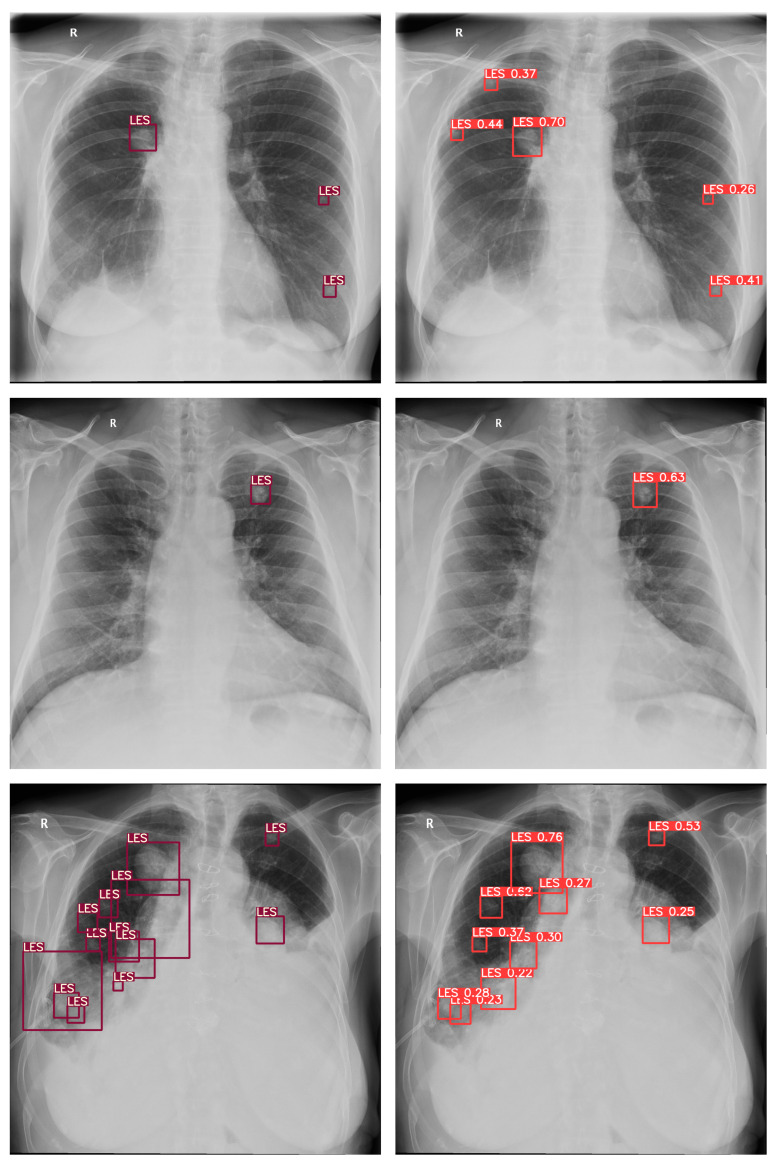
Examples of the respected ground truth pixel-level annotations and correct DLAD predictions (TP). The proposed DLAD correctly identified 91 out of 100 CXRs (*Se* of 0.910 (0.854–0.966)) pulmonary lesions (LES+ Abnormal).

**Figure A1:**
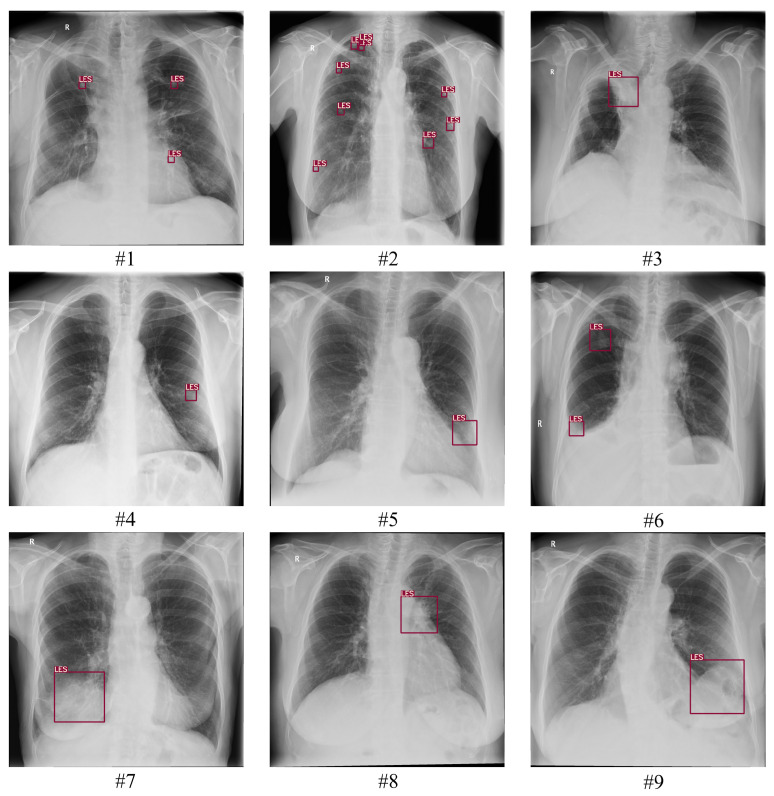
False negative (FN) images incorrectly classified by DLAD software during the retrospective study as true negative (TN) and the respected ground truth pixel-level annotation. Assessed radiologist RAD 1 also incorrectly classified CXR images #1, #2, #4, #5, #6, #7, #8 and #9. Assessed radiologist RAD 2 also incorrectly classified CXR images #1, #2, #3, #4, #5, #6 and #8. Assessed radiologist RAD 3 also incorrectly classified CXR images #1, #2, #3, #4, #5, #6 and #8. Assessed radiologist RAD 4 also incorrectly classified CXR images #1, #2, #4, and #8. Assessed radiologist RAD 5 also incorrectly classified CXR images #2, #4, #5, #6 and #9.

**Figure A2:**
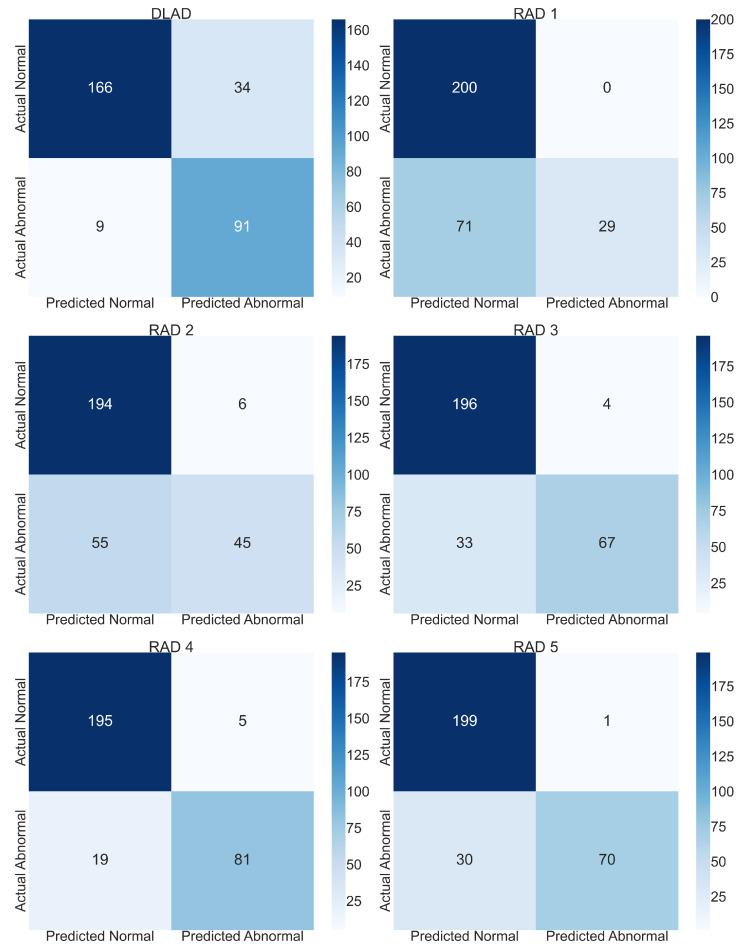
Confusion matrix showing the performance of the proposed DLAD and individual radiologists. All of them incorrectly evaluated some assessed LES+ Abnormal CXRs as without suspicious lesions. For individual radiologists, it was 62, 46, 24, 10 and 21 images, which the assessed radiologists would check again and/or consult with a more experienced colleague.

A total of 300 images with established ground truth were evaluated, of which 100 (55 ♀, 45 ♂) were from patients with one or more confirmed pulmonary lesions (LES+ Abnormal), 100 (68 ♀, 32 ♂) were from patients with various confirmed findings other than pulmonary lesions (LES− Abnormal), and 100 (79 ♀, 21 ♂) were without any pathological findings (Normal). The proposed deep-learning-based automatic detection algorithm (DLAD) correctly identified 91 out of 100 CXRs (*Se* of 0.910 (0.854–0.966), Figure 4) with pulmonary lesions (LES+ Abnormal) and 165 out of 200 CXRs (*Sp* of 0.815 (0.761–0.900)) as either without pulmonary lesions (LES− Abnormal) or without any abnormalities (Normal). There were 45 (15%) images that were incorrectly classified as having pulmonary lesions, even though they did not, 26 of which were originally determined as Abnormal LES− and 19 as Normal, indicating that DLAD was slightly more likely to encounter difficulties with comorbid patients than with patients without any abnormalities. In particular, rib summation and more prominent pulmonary vascular markings were among the problematic regions that indicated multiple false positives (FP). The higher false positive rate of the proposed DLAD was expected because the threshold was set to classify even suspect findings as abnormal. Additionally, nine (3%) images with pulmonary lesions were incorrectly classified as not having any lesions (FN). The false-negative CXR images with respected ground truth pixel-level annotations and confusion matrices for the DLAD and individual radiologists can be found in the Appendix A (Figure A1 and Figure A2).

**Figure 5:**
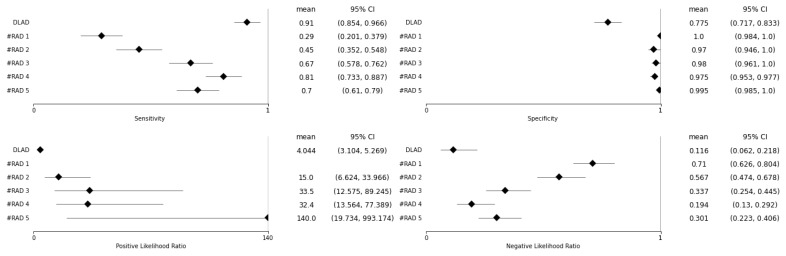
Forest plots showing the mean sensitivity (*Se*), specificity (*Sp*), positive *(PLR)*, and negative likelihood ratio *(NLR)* and corresponding 95% confidence interval estimates for DLAD and individual radiologists. For all assessed radiologists, the DLAD achieved a statistically significantly higher *Se* than that of radiologists, indicating that it would be useful in identifying patients with pulmonary lesions that were not identified by the radiologists.

For all assessed radiologists, the proposed DLAD achieved a statistically significantly higher *Se* (0.910 (0.854–0.966)) than that of radiologists (RAD 1: 0.290 (0.201–0.379), *p* < 0.001; RAD 2: 0.450 (0.352–0.548), *p* < 0.001; RAD 3: 0.670 (0.578–0.762), *p* < 0.001; RAD 4: 0.810 (0.733–0.887), *p* = 0.025; RAD 5: 0.700 (0.610–0.790), *p* < 0.001), and would therefore help to identify patients with pulmonary lesions that the radiologists evaluated as being without the suspicious findings (Table 3, Figure 5). However, the DLAD achieved a lower *Sp* (0.775 (0.717–0.833)) than all assessed radiologists (RAD 1: 1.000 (0.984–1.000), *p* < 0.001; RAD 2: 0.970 (0.946–1.000), *p* < 0.001; RAD 3: 0.980 (0.961–1.000), *p* < 0.001; RAD 4: 0.975 (0.953–0.997), *p* < 0.001; RAD 5: 0.995 (0.985–1.000), *p* < 0.001), and the difference was statistically significant. As no images were evaluated as FP by RAD 1, the *CI* was calculated using the Clopper–Pearson method.

The DLAD PLR (4.044 (3.104–5.269)) was significantly lower (i.e., worse) than that of radiologists (RAD 1: N/A (no CXR was evaluated as FP), RAD 2: 15.000 (6.624–33.966), *p* = 0.002; RAD 333.500 (12.575–89.245), *p* < 0.001; RAD 4: 32.400 (13.564–77.389), *p* < 0.001; RAD 5: 140.000 (19.734–993.174), *p* < 0.001); the NLR (0.116 (0.062–0.218)) was lower (i.e., better) than that of radiologists (RAD 1: 0.710 (0.626–0.804), *p* < 0.001; RAD 2: 0.567 (0.474–0.678), *p* < 0.001; RAD 3: 0.337 (0.254–0.445), *p* < 0.001; RAD 4: 0.194 (0.130–0.292), *p* = 0.132; RAD 5: 0.301 (0.223–0.406), *p* = 0.003); and the difference was statistically significant, with the exception of RAD 4, where no statistical difference was found for this parameter (*p* > 0.05). (Table 4, Figure 5).

## 5. Discussion

The present retrospective study investigated the performance of a deep learning-based automatic detection algorithm (DLAD) for the detection of pulmonary lesions on chest X-ray (CXR) data from a specialized oncology center. The proposed DLAD demonstrated higher sensitivity (*Se* 0.910 (0.854–0.966)) than all five assessed radiologists: RAD 1: 0.290 (0.201–0.379), *p* < 0.001; RAD 2: 0.450 (0.352–0.548), *p* < 0.001; RAD 3: 0.670 (0.578–0.762), *p* < 0.001; RAD 4: 0.810 (0.733–0.887), *p* = 0.025; RAD 5: 0.700 (0.610–0.790), *p* < 0.001). The effectiveness was indirectly demonstrated by its ability to alert to the presence of a pulmonary lesion that a doctor might have missed. While the proposed DLAD may produce more false positive results (*Sp* 0.775 (0.717–0.833) than radiologists (RAD 11.000 (0.984–1.000), *p* < 0.001, RAD 20.970 (0.946–1.000), *p* < 0.001, RAD 30.980 (0.961–1.000), *p* < 0.001, RAD 40.975 (0.953–0.997), *p* < 0.001, RAD 50.995 (0.985–1.000), *p* < 0.001), its ability to identify overlooked lesions makes it a useful decision-support tool in clinical practice. For individual radiologists, there were 62, 46, 24, 10, and 21 CXR images with missed suspicious lesions that the assessed radiologists would re-evaluate and/or seek guidance on from experienced colleagues.

Compared with related studies that applied a similar methodology, the proposed DLAD model achieved high sensitivity on a relatively large and well-described dataset. The trade-off between *Se* and *Sp* was expected, as the DLAD was designed to serve as a decision-support system. The results of this study are largely unique because they investigated detection performance on retrospectively collected data from a specialized cancer center where the prevalence of patients with suspicious lung lesions may be higher. Comparable results were presented in [22], where the performance of a commercial DLAD was investigated in a multi-centric study. The investigated DLAD demonstrated *Sp* of 0.952 while preserving high *Se* of 0.807, whereas the averaged *Se* of the physicians was 0.704. Ref. [24] evaluated the performance of a commercial DLAD to detect five findings on CXRs including pulmonary nodules, but the test data contained only 24 images with suspicious lesions. The proposed DLAD reached an AUC of 0.773 for pulmonary lesion detection, which, given the similar ground truth approach as in our study, is a considerably worse score. An interesting approach was proposed in [25], which simulated clinical practice with a high prevalence of patients with suspicious lesions (600 CXRs with nodules, 200 normal CXRs). The DLAD was compared to 12 radiologists and achieved *Se* of 0.86 compared to the radiologists’ 0.79. However, it must be stated that the prevalence of patients with suspicious lesions in clinical practice is significantly lower [35,36]. The comparison with other related studies was impossible due to the different evaluation methodologies: in [23], radiologists evaluated original CXRs and BSI CXRs, [26] did not present *Se* and *Sp* values but only an increase in accuracy, and [27] did not compare performance against physicians.

### Limitations

While DLADs can provide valuable assistance to radiologists in detecting pulmonary lesions on CXR images, their use also carries potential risks and harm to patients. One of our main concerns with the use of the proposed DLAD in medical diagnosis is the potential for incorrect or misleading results. Since the algorithm is based on statistical models, it can make mistakes or produce false positives and negatives, leading to incorrect diagnoses. This can have consequences for patients, who may be given unnecessary treatments or miss out on vital care [37]. The false-positive rate of the proposed DLAD was higher than that of the comparison radiologists. However, it should be added that differential diagnosis or confusion with normal human-body factors played a role. Furthermore, there is a risk that DLAD may be trained on a dataset that is not representative of the general population, leading to unequal or unfair treatment of certain groups of patients [38]. This can have profound implications for patient care and can result in discrimination and unequal access to medical services. Since this study was conducted retrospectively and the assessed radiologists did not have access to patients’ clinical information, it may not accurately reflect real-world conditions. These factors can be minimized by carrying out a prospective multi-centric study or by retrospectively evaluating large prospectively collected multi-institutional datasets in simulated clinical practice. In addition, the option of supporting ground truth with other diagnostic methods, such as biopsy or longer-term follow-up of the lesion, should be explored. While this study showed promising results achieved with a relatively large sample of patients, further clinical validation process needs to be conducted to determine the applicability of the proposed DLAD.

## 6. Conclusions

The purpose of this study was to analyze the performance of a deep learning-based automatic detection algorithm (DLAD, Carebot AI CXR v2.00) in identifying suspicious pulmonary lesions on CXR, and to compare its accuracy to that of five individual radiologists. The proposed DLAD demonstrated improved detection performance compared to existing conventional imaging-based diagnostics, as it showed a significantly lower false-negative rate while also providing relatively high specificity.

## Figures and Tables

**Table 1:** Methodology of determining the ground truth by assessment of three radiologists with different levels of experience.

Class	Inclusion Criteria
**LES+ Abnormal**	A consensus of 2/3 is required to confirm the presence of one or more pulmonary lesions. In addition, CXR may contain other pathological abnormalities. A consensus of 2/3 is required to confirm these.
**LES− Abnormal**	A consensus of 3/3 is required to confirm the absence of any pulmonary lesions. In addition, CXR contained other pathological abnormalities. A consensus of 2/3 is required to confirm these.
**Normal**	A consensus of 3/3 is required to confirm the CXR did not show any pathological abnormalities.

**Table 2:** Patient characteristics indicating the demographic data and the prevalence of individual findings.

Data	LES+ Abnormal	LES− Abnormal	Normal
**Total**	100	100	100
**Patient’s Sex**			
Female (♀)	55	68	79
Male (♂)	45	32	21
F:M Ratio	1.22:1	2.13:1	3.76:1
**Prevalence**			
LES only	40	0	0
With other findings	60	100	0
**Findings**			
Atelectasis	5	6	0
Consolidation	21	21	0
Cardiomegaly	5	38	0
Fracture	1	10	0
Mediastinal widening	1	0	0
Pneumoperitoneum	0	1	0
Pneumothorax	0	0	0
Pulmonary edema	0	9	0
Pleural effusion	21	34	0
Pulmonary lesion	100	0	0
Hilar enlargement	2	2	0
Subcutaneous emphysema	0	1	0

**Table 3:** Pooled results of DLAD and individual radiologists sensitivity (*Se*) and specificity (*Sp*) with corresponding 95% confidence interval estimates. The experience levels of the radiologists were as follows: RAD 1 and RAD 2 were junior radiologists with less than 2 years of experience, RAD 3 was a radiologist with more than 2 years but less than 5 years of experience, and RAD 4 and RAD 5 were board-certified radiologists with more than 5 years of experience.

	BA	*Se* (95% CI)	*Sp* (95% CI)	*Se p*-Value	*Sp p*-Value
**DLAD**	**0.843**	**0.910 (0.854–0.966)**	**0.775 (0.717–0.833)**		
**RAD 1**	0.645	0.290 (0.201–0.379)	1.000 (0.984–1.000)	<0.001	<0.001
**RAD 2**	0.710	0.450 (0.352–0.548)	0.970 (0.946–0.994)	<0.001	<0.001
**RAD 3**	0.825	0.670 (0.578–0.762)	0.980 (0.961–1.000)	<0.001	<0.001
**RAD 4**	0.893	0.810 (0.733–0.887)	0.975 (0.953–0.997)	0.025	<0.001
**RAD 5**	0.848	0.700 (0.610–0.790)	0.995 (0.985–1.000)	<0.001	<0.001

**Table 4:** Pooled results of DLAD and individual radiologists likelihood ratio (*LR*) with corresponding 95% confidence interval estimates. The experience levels of the radiologists were as follows: RAD 1 and RAD 2 were junior radiologists with less than 2 years of experience, RAD 3 was a radiologist with more than 2 years but less than 5 years of experience, and RAD 4 and RAD 5 were board-certified radiologists with more than 5 years of experience.

	PLR	NLR	PLR *p*-Value	NLR *p*-Value
**DLAD**	**4.044 (3.104–5.269)**	**0.116 (0.062–0.218)**		
**RAD 1**	N/A	0.710 (0.626–0.804)	N/A	<0.001
**RAD 2**	15.000 (6.624–33.966)	0.567 (0.474–0.678)	0.002	<0.001
**RAD 3**	33.500 (12.575–89.245)	0.337 (0.254–0.445)	<0.001	<0.001
**RAD 4**	32.400 (13.564–77.389)	0.194 (0.130–0.292)	<0.001	0.132
**RAD 5**	140.000 (19.734–993.174)	0.301 (0.223–0.406)	<0.001	0.003

## Data Availability

Data from this study can be provided by Carebot, Ltd., to independent researchers. Please contact the author for more information, if required.

## Abbreviations

The following abbreviations are used in this manuscript:

AI	Artificial Intelligence
CI	Confidence Interval
CNN	Convolutional Neural Network
CXR	Chest X-ray
DL	Deep Learning
DLAD	Deep Learning–based Automatic Detection Algorithm
Se	Sensitivity
Sp	Specificity
BA	Balanced Accuracy
TP	True Positive
FP	False Positive
TN	True Negative
FN	False Negative
LR	Likelihood Ratio
PLR	Positive Likelihood Ratio
NLR	Negative Likelihood Ratio
PV	Predictive Value
PPV	Positive Predictive Value
NPV	Negative Predictive Value
